# Problem-Based Learning to Encourage Active Learning and Teamwork Among First Year Medical Students - Student Reports in 2020 - What is the Best Response When Listeners Fail to Hear English Correctly? (Course name: Listening Skills: Development and Assessment)

**DOI:** 10.14789/jmj.JMJ21-0054-OT

**Published:** 2022-11-18

**Authors:** MOKAN KAKU, MIREI SHIROYANAGI, HARUHI MIZUMA, RYOKO FUJITA

**Affiliations:** 1 School of Medicine, Juntendo University, Tokyo, Japan; 1 School of Medicine, Juntendo University, Tokyo, Japan; 2 Department of General Education, Juntendo University Faculty of Medicine, Chiba, Japan; 2 Department of General Education, Juntendo University Faculty of Medicine, Chiba, Japan

**Keywords:** seeking clarification, response, communication strategy, survey

## Abstract

**Objective:**

This study was aimed at investigating the best way to seek clarification when failing to correctly understand the meaning of an English utterance.

**Materials and Methods:**

We administered a survey to Japanese freshmen majoring in medicine to determine the main reasons for their failure to hear utterances and how they seek clarification when facing different situations of mishearing. The same survey was distributed to four teachers who are native English speakers.

**Results:**

Generally, the students obtained a low percentage of correct answers to multiple-choice questions. The use of a particular test-taking strategy depended primarily on an individual, but the students evaluated all the choices when answering multiple-choice questions.

**Conclusions:**

The current study revealed that the most common causes of mishearing among the participants were a lack of vocabulary and speech speed. The most popular strategy for seeking clarification was asking for it directly. Finally, the native speakers and highly proficient students tended to attempt to understand utterances indirectly.

## Introduction

Seeking clarification is one of the vital skills that language learners need to acquire. Compared with native speakers, language learners encounter many occasions where they cannot understand what the other people are saying. The lack of understanding comes from various reasons, such as insufficient vocabulary knowledge, limited cultural knowledge, or simply the fast speech rate. A member of our team is an international student who understands the difficulty of learning a foreign language. Adapting to life in a foreign country necessitates the ability to seek clarification when failing to understand the meanings of utterances. Even when there are no language barriers, we need to seek clarification in many occasions. For example, during lectures, an unavoidable problem for the students is that students sometimes fail to hear an utterance correctly owing to a loss of concentration^1)^. If we cannot understand what the others are saying, various problems arise. For language learners, they cannot communicate with others. For students, they cannot learn what they are supposed to.

For the reasons stated above, we think this issue is an interesting topic for exploration. Correspondingly, this research investigated the best way to seek clarification by administering questionnaires to Japanese students and four teachers (three from the US and one from Canada) who are native English speakers.

## Literature review

The purpose of the current research is to inquire into the most effective strategies for seeking clarification in situations where utterances are misheard or misunderstood and to examine how native and non-native speakers of English use different strategies for seeking clarification.

Mizuta^2)^ examined language learners' use of strategies in listening to a lecture. The participants were Chinese students who studied Japanese. They listened to a lecture in Japanese and reported their listening comprehension process. Based on the results, Mizuta classified the reasons for mishearing into external distractions, a lack of recognition, a lack of vocabulary, and a lack of understanding of connections. External distraction is related to listeners losing focus, and a lack of recognition refers to the failure to understand speech sounds. A lack of vocabulary means listeners may encounter problems understanding certain words, and a lack of understanding of connections pertains to listeners' comprehension of the meanings of words or phrases but not the link between these within a sentence.

Tsubaki^3)^ conducted a study about teaching communication strategies. The participants were international students who studied Japanese at a university in Japan. The participants received instruction about how to use communication strategies in conversations. The results showed that half of the participants acquired the skills of asking back after the training. In her study, Tsubaki advocated a theory that posited five patterns of seeking clarification. These patterns are requesting to repeat an utterance, requesting for explanation, repeating unknown words, checking for understanding, and attempting to understand utterances indirectly. A request to repeat an utterance means asking someone to restate a sentence, while a request for an explanation means asking for elaboration. Repeating unknown words involves restating a word that is unfamiliar to a listener, and checking for understanding means verifying if meaning is comprehended by asking a question. Finally, attempting to understand utterances indirectly entails grasping a word or phrase through some other known information. Clarification questions play a significant role to attain mutual understanding in spoken dialogue systems. Gabsdil^4)^ defines the term clarification question or clarification request as the questions that speakers ask when they do not fully understand or is uncertain about what the previous speaker said or meant with an utterance. According to Schlangen^5)^, clarification requests vary in their form and their function. Clarification requests take various forms, such as conventional forms, full sentences and sentential fragments. Regarding functions of clarification request, they include acoustic understanding and possibility. Linguistic differences between English and Japanese should also be mentioned. Modern English is an analytic language that primarily conveys relationships between words in sentences through particles, prepositions, and similar components^6)^. For example, “laugh on” and “laugh at” carry varying meanings, given the change in prepositions. By contrast, Japanese is an agglutinative language, in which differences in morphemes comprising words determine their meanings^7)^.

Based on the past studies stated above, seeking clarification is a vital skill that language learners need to acquire. Also, comparing native speakers' ways to seek clarification with those of language learners give important suggestions to language learners who need to improve their communication skills in target language.

The following are the research questions (RQs) for the current study.

RQ1: What are the main reasons for language learners' failure to hear utterances?

RQ2: How are language learners seek clarification when facing different situations of mishearing?

RQ3: Are the ways language learners seek clarification different from those used by native speakers?

Regarding the way language learners seek clarification, we hypothesized that attempts at indirect understanding would be the favored strategy. Tsubaki^3)^ argued that endeavors to understand utterances indirectly do not change the flow of Japanese conversations. Therefore, we hypothesized that this idea also applies to English conversations.

## Method

The purpose of the current study was to examine how Japanese learners of English seek clarification in conversations and compare the strategies used by native speakers of English with those used by language learners. We conducted a questionnaire survey to both university students and instructors using Google Form.

### Participants

Sixty-eight Japanese university students participated in the study. They are all first-year students majoring in medicine. Their proficiency levels ranged from CEFR A2 to B2 based on the placement test they took after entering the university. Four faculty members of a university also took part in the study. We needed responses from native speakers of English, but because of the time constraint, the best way to get responses from native speakers was to ask questions to the instructors at the university that we attended. Three of the instructors came from the US and one was from Canada.

### Materials

We used the questionnaire because of two reasons. The first reason is that questionnaire suited the best for the purpose of the study. As the purpose of the study was to examine how language learners seek clarification in real-life communication, the best way to get the participants' responses was to ask them to think back the time when they had communication problems, and choose their preferences. The second reason is the COVID pandemic. As the study was conducted during the COVID pandemic, it was difficult to have in-person interviews or conduct tests to the participants in person. We developed a questionnaire and distributed it to the respondents. It consisted of two questions (see Appendix). Question 1 was intended to determine the main reasons for the participants' failure to hear English utterances correctly. Question 2 was designed to ascertain how the participants seek clarification when facing different situations of mishearing.

Question 2 was comprised of four parts. Questions 2a and 2b were meant to discover how participants seek clarification when they talk to people close to them. The first response option is requesting repetition, the second is requesting an explanation, the third is repeating unknown words, the fourth is checking for understanding, and the fifth is attempting to understand utterances indirectly. Questions 2c and 2d are the same, except that the situations involve individuals who are not close to the respondents.

### Procedure

We distributed the questionnaire to the participants. We used Google Form and sent the participants the URL of the Google Form. It took about 5 to 10 minutes for the participants to finish answering the questionnaire.

## Results

**Figure 1:**
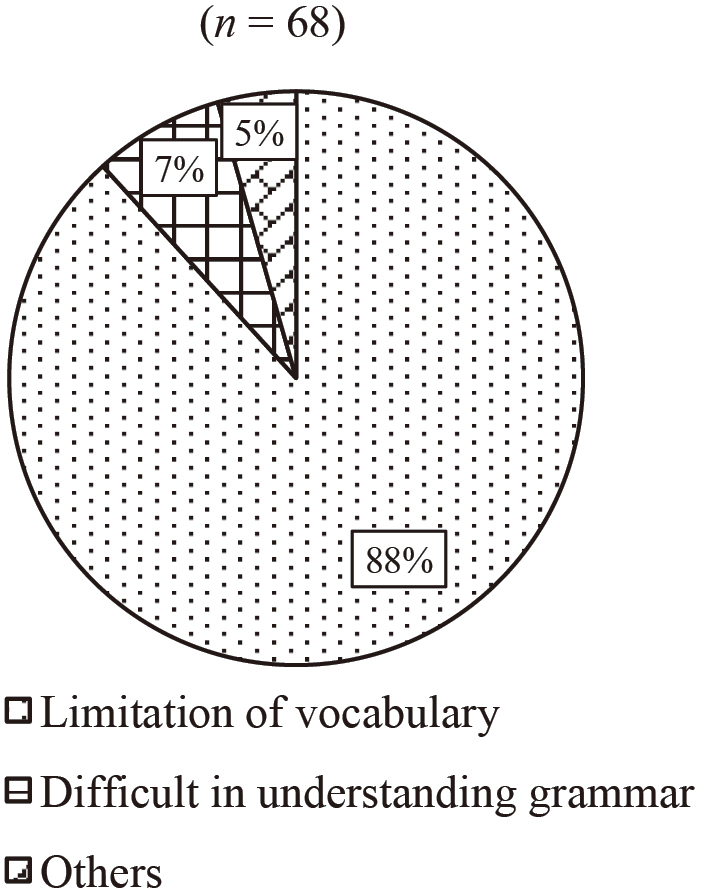
Question 1: What is the major reason why you fail to hear? Q1a: When you cannot understand content

**Figure 2:**
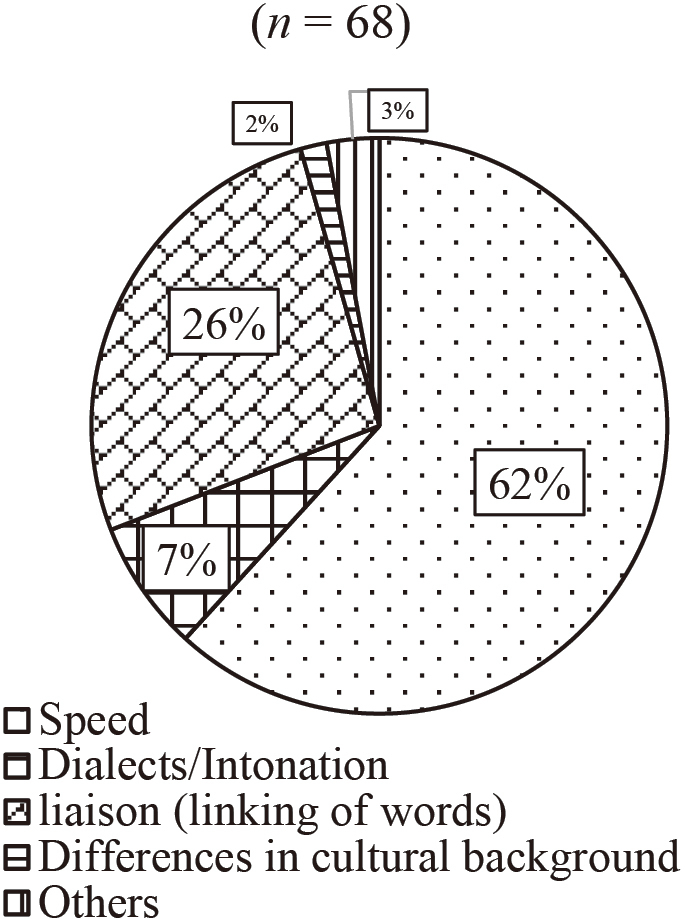
Question 1: What is the major reason why you fail to hear? Q1b: When you can understand a sentence but not in dialogues

The questionnaire survey was conducted for the current study to investigate the main reasons of language learners' failure to hear utterances and how they seek clarification when facing different situations of mishearing. The responses of language learners were also compared with those of native speakers of the target language. Figures 1 and 2 present the results of Question 1 (What is the major reason why you fail to hear?). Figure 1 shows the results of Q1a (When you cannot understand content). The majority of them (88 %) answered that the major reason was due to the limitation of vocabulary. Figure 2 shows the results of Q1b (When you can understand a sentence but not in dialogues). 62 % of them answered it is because of the spend, and 26 % answered that because of liaison.

**Figure 3:**
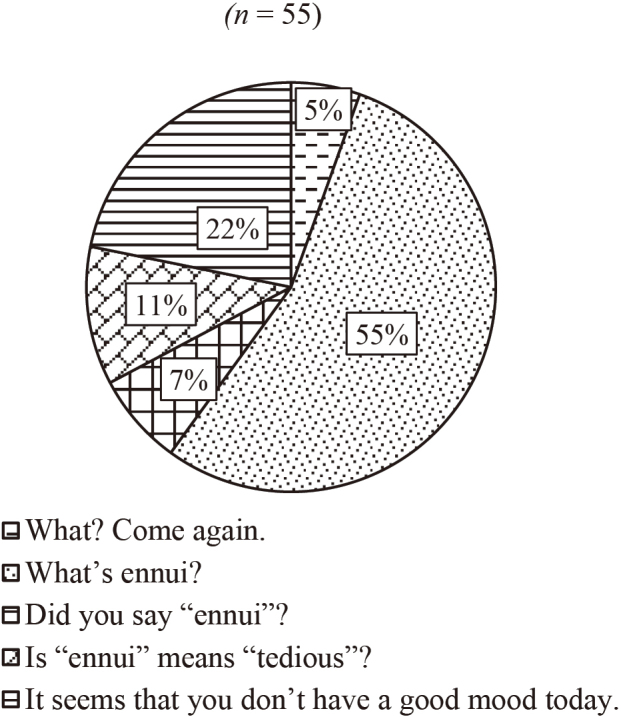
Question 2: What do you think is the best response? Q2a: To a best friend

**Figure 4:**
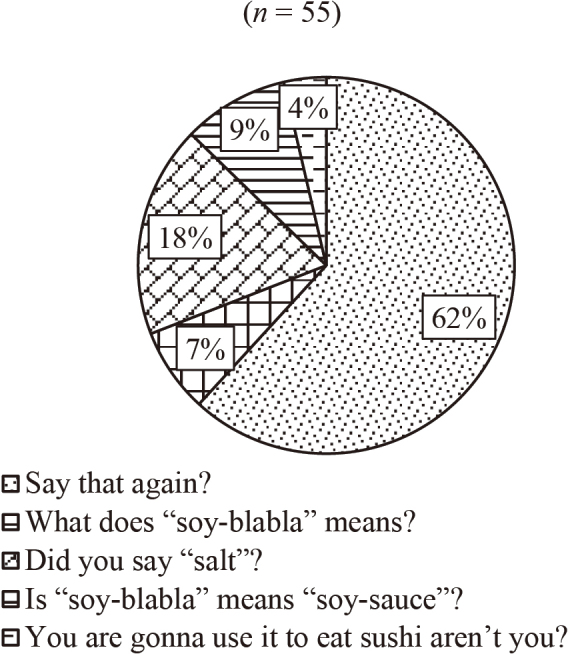
Question 2: What do you think is the best response? Q2b: To a family member

**Figure 5:**
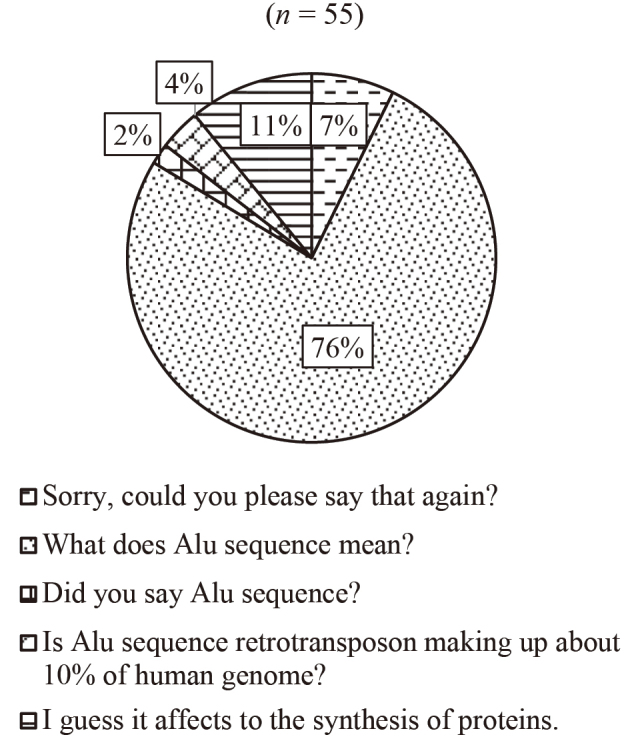
Question 2: What do you think is the best response? Q2c: To an older person (Teacher)

**Figure 6:**
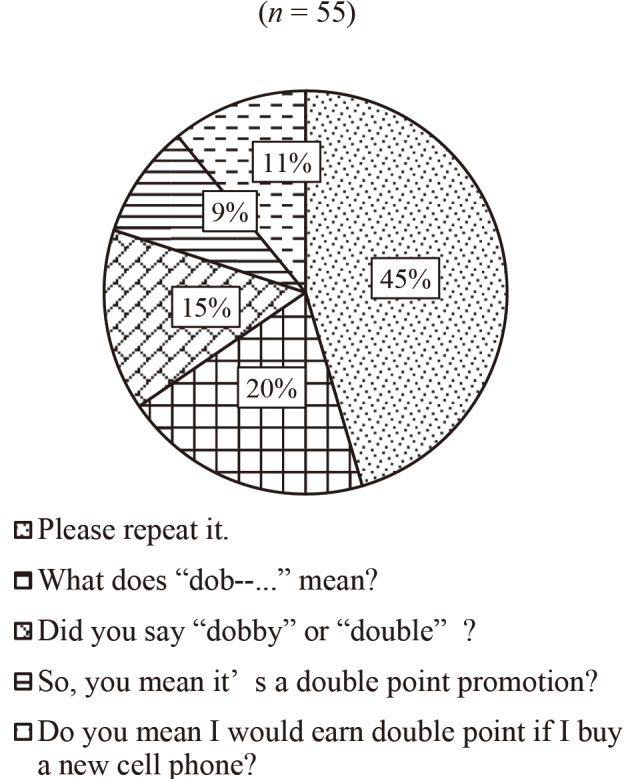
Question 2: What do you think is the best response? Q2d: To an unknown person (Salesclerk)

Figures 3 to 6 show the answers to Question 2 (What do you think is the best response?). In Question 2, there are four questions from Q2a to Q2d, which are divided into two categories, talking to familiar people (Q2a and Q2b) and unfamiliar people (Q2c and Q2d).

Figure 3 shows the results of the best response when talking to a best friend. About half of the participants chose “What's ‘ennui'?” And 22 % of them chose repeating, “Did you say ‘ennui'?” As shown in Figure 4, 62 % of the respondents chose “Say that again?” when talking to a family member. When they talk to an older person, such as a teacher, the most popular response (79%) was “Sorry, could you please say that again?” (see Figure 5). Figure 6 shows the case of talking to an unknown person, such as a salesclerk. The students' responses varied. 45 % of them chose “Please repeat that.” And 20 % chose “What does ‘dob …' mean?”

We also asked the same question items to the four instructors whose native tongue was English. For Question 2a, talking to a best friend, all the teachers chose “What's ‘ennui'?” as their response, and for Question 2b, talking to a family member, three of them selected “Say that again?”, whereas one chose “What does ‘soy-blabla' mean?” as their answers. For Question 2c, talking to an older person, two teachers chose “What does ‘Alu sequence' mean?”, one selected “In an Alu sequence, does retrotransposon make up about 10% of the human genome?”, and one opted for “Sorry, could you please say that again?” as their responses. With regard to Question 2d, talking to an unknown person, two teachers opted for “Please repeat that.” as a strategy for seeking clarification, and two chose “So, you mean it's a double point promotion?”.

## Discussion

### RQ1: What are the main reasons for language learners' failure to hear utterances?

The findings related to Question 1 showed that failure among the respondents to hear and understand content was most frequently caused by limitations in vocabulary. These results support what Mizuta^2)^ argued. According to Mizuta, a lack of vocabulary is one of the reasons why language learners mishear in their listening comprehension process. Speed was the most common cause of the participants' failure to hear understandable sentences. These results point to the importance of being careful with these two aspects so that people can correctly hear utterances.

### RQ2: How are language learners seek clarification when facing different situations of mishearing?

The most popular answers to Question 2 were “request to repeat” and “request to explain” in various situations of mishearing. Both can be classified as instances of direct asking, which contrasts with indirect asking, such as attempts to understand utterances indirectly and checking for understanding.” We initially hypothesized that attempts at indirect understanding would be the favored strategy. This assumption was promoted by Tsubaki^3)^, who asserted that endeavors to understand utterances indirectly do not change the flow of Japanese conversations. We speculated that this idea is also applicable to English conversations. The results negated our hypothesis.

Some of the Japanese participants could not ask for clarification in an indirect manner because English and Japanese are different languages. As the structure of English is different from that of Japanese, some Japanese learners' insufficient language skills prevent them from asking for clarification in an indirect manner.

The choices made by the Japanese non-native students and native English teachers were compared. In all the cases (Questions 2a-2d), the most frequent choices made by the students were identical to those made by the teachers. That is, both groups tended to choose “asking directly” as a response. This finding suggests that whether listeners are native or non-native English speakers does not matter considerably.

The comparison of the results on two types of failure to hear (able to hear but could not understand meaning and able to understand but could not recognize an utterance) uncovered certain trends. For the most frequently occurring failure—the ability to understand accompanied with the inability to recognize an utterance—the most popular solution was to request repetition. For the other type of failure, the most favored strategy was to request an explanation. These findings are logical because the chosen strategies are the shortest way to solve the corresponding problems. People change their approaches depending on what a problem is.

For the other type of failure—the ability to understand accompanied with the inability to recognize an utterance—the second common strategy was attempting to understand utterances indirectly. These results accord with the argument suggested by Schlangen^5)^. Schlangen stated that clarification request vary in their form and their function.

### RQ3: Are the ways language learners seek clarification different from those used by native speakers?

In order to compare the way language learners and native speakers seek clarification, we asked four faculty members whose native language was English. We observed some similarities and differences between the students and native speakers. About Question 2a, talking to a best friend, all of the native speakers chose the direct way of asking, “What's ‘ennui'?” In case of the students, even though half of them chose the same option, repeating, “Did you say ‘ennui'?” was the second most popular response. About Question 2c, talking to an older person, most of the students chose “Sorry, could you please say that again?” However, the responses from native speakers varied. Two of them chose a direct way, “What does ‘Alu sequence' mean?” Only one native instructor chose “Sorry, could you please say that again?”

We also observed some differences in the students' responses depending on their language proficiency levels. Among those who chose the strategy of attempting to understand utterances indirectly as answers to Questions 2b and 2d, approximately 60% to 70% were from English classes A and B—classes attended by high-level English users. It can be hypothesized that individuals who can handle English better tend to choose indirect understanding, as this strategy requires high English proficiency. The differences between native speakers and language learners, as well as those between high-proficient and low-proficient learners could be due to linguistic differences. As stated in the past studies, English is an analytic language conveying relationships between words in sentences through particles, prepositions, and similar components^6)^. Compared to English, the meanings in Japanese are determined by morphemes. As English speakers try to find relationships between words, they might opt for indirect understanding.

## Conclusion

The study's findings are summarized as follows: First, the most common causes of hearing failure were a lack of vocabulary and speech speed. Second, “asking directly” was the most popular strategy for seeking clarification. Note, however, that the native speakers and highly proficient students tended to favor indirect understanding. No clear patterns were found when the fellow interactant of a speaker was different, familiar versus unfamiliar. In the future, more accurate conclusions would be obtained using more randomized and larger samples.

## Funding

No funding was received.

## Author contributions

MK, MS, and HM planned the work and wrote the manuscript. RF supervised the work and revised the manuscript. All authors read and approved the final manuscript.

## Conflicts of interest statement

The authors have declared that no conflicts of interest exist.

## Supplementary Material

**Appendix:**
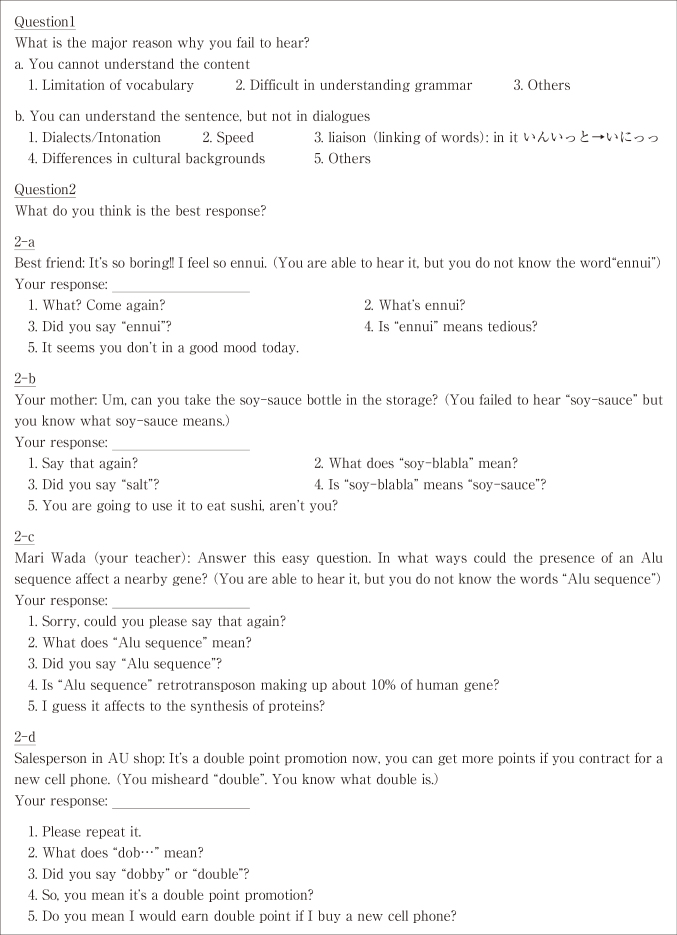
The questionnaire distributed to the respondents
